# Effects of Alcohol Compounds on the Growth and Lipid Accumulation of Oleaginous Yeast *Trichosporon fermentans*

**DOI:** 10.1371/journal.pone.0046975

**Published:** 2012-10-05

**Authors:** Chao Huang, Hong Wu, Li-ping Liu, Wen-yong Lou, Min-hua Zong

**Affiliations:** 1 State Key Laboratory of Pulp and Paper Engineering, College of Light Industry and Food Sciences, South China University of Technology, Guangzhou, PR China; 2 Key Laboratory of Renewable Energy and Gas Hydrate, Guangzhou Institute of Energy Conversion, Chinese Academy of Sciences, Guangzhou, PR China; 3 Laboratory of Applied Biocatalysis, College of Light Industry and Food Sciences, South China University of Technology, Guangzhou, PR China; Yonsei University, Republic of Korea

## Abstract

The inhibitors present in dilute acid-treated lignocellulosic hydrolysates would show great effect on the growth and product formation of microorganisms. To understand their inhibitory law and mechanism on oleaginous microorganism could help improving the efficiency of lignocellulose hydrolysis, detoxification, and lipid fermentation. The effects of four representative alcohol compounds present in lignocellulosic hydrolysates, including furfuryl alcohol, vanillyl alcohol, catechol, hydroquinone on the cell growth and lipid accumulation of *Trichosporon fermentans* were systematically investigated in this work. The toxicity of selected alcohol compounds was well related to their log *P* value except furfuryl alcohol, whose log *P* value was the minimum but with the highest toxicity to *T. fermentans*. The inhibition of all the alcohol compounds on the growth of *T. fermentans* was more serious than on the lipid synthesis. Also, the growth of *T. fermentans* was more sensitive to the variation of inoculum size, temperature, and initial pH than lipid synthesis in the presence of alcohol compounds. Initial pH had more profound influence on the lipid fermentation than inoculum size and cultural temperature did. Careful control of fermentation conditions could be helpful for improving lipid yield of *T. fermentans* in lignocellulosic hydrolysates. Among the four alcohol compounds tested, most alcohol compounds showed inhibition on both sugar consumption and malic enzyme activity of *T. fermentans*. However, vanillyl alcohol had little influence on the malic enzyme activity. Similarly, all alcohol compounds except vanillyl alcohol exerted damage on the cell membrane of *T. fermentans*.

## Introduction

Biodiesel is one of potential alternatives to traditional petrodiesel oil [1], however, the high cost of its lipid feedstock unfortunately prevents its industrialization process. On the other hand, using vegetable oils for biodiesel production might bring the “food vs. fuel” debate. Albeit using waste oils could reduce the cost of lipid feedstock, their limited provision is another problem [2]. Hence, microbial oils, namely single cell oils (SCOs), whose composition are similar to those of vegetable oils, are believed to be the promising raw materials for biodiesel production because of their many advantages such as the fast growth rate of microorganisms, and facile production unaffected by the place, climate, and season [3]. In spite of the favorable impacts SCO might exert, the economic aspect of current SCO production has been restricted primarily by the high medium cost [4]. The utilization of inexpensive substrates, such as agro-industrial residues, especially lignocellulosic materials, the most abundant and renewable biomass resources in the world for fermentation is one of practical strategies to solve this problem.

**Figure 1:**
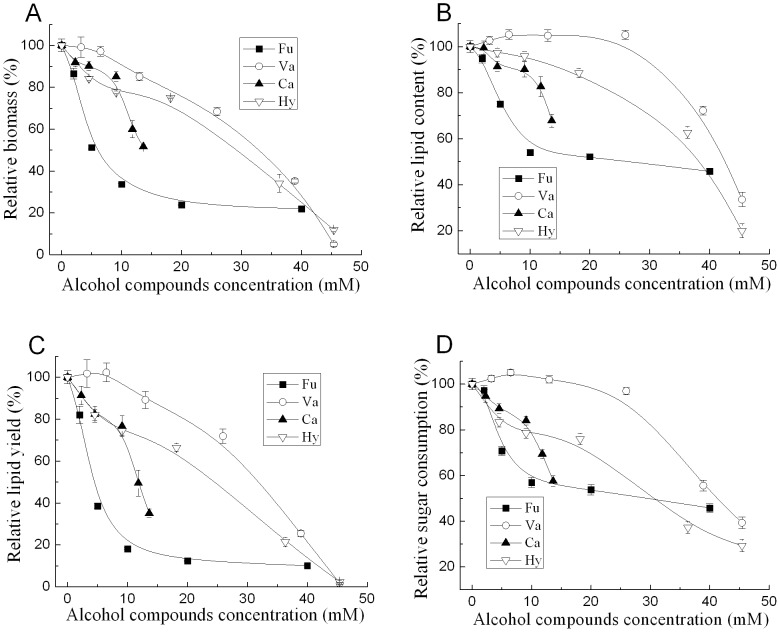
Effect of selected alcohol compounds on the growth and lipid accumulation of *T. fermentans*. (A) Biomass; (B) lipid content; (C) lipid yield; (D) sugar consumption. The biomass, lipid content, lipid yield, and sugar consumption of *T. fermentans* were 24.0 g/L, 61.7%, 14.8 g/L, and 84.3 g/L at the 7^th^ day in the medium without inhibitors. Abbreviations: Ca, catechol; Hy, Hydroquinone; Fu, Furfuryl alcohol; Va, Vanillyl alcohol.

The possibility of using lignocellulosic materials for SCO production has been proven by many works [5], [6]. However, the growth and lipid accumulation of oleaginous microorganism might be influenced by different lignocellulosic hydrolysates due to their complex composition. Generally, besides sugars, various by-products so called “inhibitors” are inevitably generated during the dilute acid hydrolysis or pretreatment process [7] and these compounds could affect the growth and product formation of microorganisms during fermentation. The effect of inhibitors in lignocellulosic hydrolysates on the growth and lipid accumulation of oleaginous microorganisms has been studied by some researchers, however, most of these works mainly focused on the effect of aldehydes and organic acids [8], [9]. Also, in our previous studies on the influence of organic acids [10] and aldehydes [11], we found that these two kinds of compounds showed different effect on the growth and lipid accumulation of *T. fermentans*.

Besides organic acids and aldehydes, various alcohol compounds are also generated during the pretreatment or hydrolysis process of lignocellulosic biomass, and most of them are the products of the degradation of lignin [12]. It is well known that the inhibitory mechanism of alcohol compounds on the ethanologenic yeasts basically includes the inhibition of the sugar metabolism and the damage to the cell membrane integrity [7], [12]. However, to date, there is no systematical study focused on the effect of alcohol compounds on the growth and lipid accumulation of oleaginous microorganisms and the inhibitory mechanism of alcohol compounds on the oleaginous yeasts as well. Thus, in this work, we systematically investigated the inhibitory effects of four representative alcohol compounds (catechol, hydroquinone, furfuryl alcohol, and vanillyl alcohol) present in lignocellulosic hydrolysates on the growth and lipid accumulation of *T. fermentans*. Moreover, the effects of inoculum size, temperature, and initial pH on the inhibition of alcohol compounds were measured. Finally, the influence of alcohol compounds on the sugar metabolism, malic enzyme activity, cell morphology, cell membrane integrity of *T. fermentans* was investigated to understand the inhibitory mechanism of alcohol compounds on the growth and lipid accumulation of *T. fermentans*.

## Results and Discussion

### Effect of Alcohol Compound on the Growth and Lipid Accumulation of *T. fermentans*

Although the concentration of alcohol compounds is lower than that of organic acids or aldehydes, there are many of them present in lignocellulosic hydrolysates and most of them are aromatic compounds generated by the degradation of lignin in the process of hydrolysis or pretreatment of lignocellulosic biomass [12]. Among them, catechol, hydroquinone, furfuryl alcohol, and vanillyl alcohol are four representative ones and chosen to be studied in this work. In many cases, catechol and hydroquinone, whose structures were similar, were found in lignocellulosic hydrolysates. In some lignocellulosic hydrolysates, the concentration of catechol could be close to 0.5 g/L while the concentration of hydroquinone was relatively lower (e.g. about 0.02 g/L in spruce acid hydrolysate) [12]. Furfuryl alcohol and vanillyl alcohol, whose concentration lower than the former two alcohol compounds, were usually studied in many works for their similar structure compared with furfural and furoic acid or vanillin and vanillic acid [9], [13].

**Table 1:** Concentration of alcohol compounds required to inhibit the lipid yield of *T. fermentans*.

Alcohol compounds	Inhibitory concentration (mM)		Log *P* c
	IC_25_ a	IC_50_ b	
Catechol	8.0	11.8	0.88
Hydroquinone	9.1	25.0	0.59
Furfuryl alcohol	2.0	3.8	0.28
Vanillyl alcohol	20.1	31.8	0.29

a Concentration of 25% inhibition on lipid yield.

b Concentration of 50% inhibition on lipid yield.

c The log *P* data was cited from Zaldivar and Ingram (1999).

Fig. 1 depicted the effects of alcohol compounds on the cell growth and lipid accumulation of *T. fermentans*. As shown in Fig. 1A, all the alcohol compounds except furfuryl alcohol showed little inhibition on the biomass at their low concentration (<5 mM). The relative biomass of *T. fermentans* was only about 50% in the medium containing 5 mM furfuryl alcohol. Interestingly, the biomass of *T. fermentans* was not influenced much by furfuryl alcohol when its concentration was above 8 mM. In the medium containing catechol, the relative biomass of *T. fermentans* decreased fast when its concentration was higher than 10 mM and at its concentration of 20 mM, *T. fermentans* cannot grow at all. As shown in Fig. 1B, the influence of all the tested alcohol compounds on the lipid accumulation of *T. fermentans* was less serious than that on the biomass. Similarly, in the medium containing furfuryl alcohol, when the furfuryl alcohol concentration was greater than 8 mM, the variation of biomass and lipid content of *T. fermentans* was not significant (analyzed by ANOVA, *P*>0.05). Surprisingly, vanillyl alcohol could even stimulate lipid accumulation of *T. fermentans* when its concentration was less than 25 mM. Similar to the effect on the lipid accumulation of *T. fermentans*, the influence of alcohol compounds on the sugar consumption of *T. fermentans* was less than that on its growth (Fig. 1D).

The concentration of alcohol compounds required to inhibit the 25% and 50% lipid yield of *T. fermentans* was measured in Fig. 1C and summarized in Table 1, also the log *P* value was given. In the work focused on the ethanologenic bacteria, the toxicity of inhibitors usually related to its log *P* value, namely, more hydrophobic the inhibitor is, stronger inhibitory effect it has [13]–[15]. Also, it has been reported that the toxicity of aldehydes on the oleaginous yeast *Rhodosporidium toruloides* was related to their hydrophobicity [9]. However, in our other works, the toxicity of aldehydes [11] and organic acids [10] to *T. fermentans* was not related to their log *P* value. In this work, the toxicity of alcohol compounds except furfuryl alcohol was well related to the log *P* value. For furfuryl alcohol, it has the lowest log *P* value while showed the highest toxicity indicated by its IC_25_ and IC_50_. However, its inhibitory course was different from other three alcohol compounds. As shown in Fig. 1, the relative biomass, lipid content and lipid yield of *T. fermentans* decreased quickly when furfuryl alcohol concentration was less than 10 mM. In contrast, in the medium containing other alcohol compounds, the relative biomass, lipid content and lipid yield of *T. fermentans* decreased much slower. The different structure between furan and aromatic compounds might account for this. Interestingly, the toxicity of catechol was obviously higher than that of hydroquinone albeit they have similar structure (Fig. 1), which in accordance with the conclusion of previous work that substituent position influenced the toxicity of inhibitors [16].

**Table 2:** Effect of different alcohol compounds on the lipid composition of *T. fermentans.*

Alcohol compounds	Concentration (g/L)	Lipid composition of *T. fermentans* (%)				
		Palmitic acid (C16∶0)	Linoleic acid (C18∶2)	Oleic acid(C18∶1)	Stearic acid (C18∶0)	Others
Vanillyl alcohol	1.0	20.8	4.6	59.5	13.3	1.8
	2.0	20.1	3.8	61.3	12.2	2.6
	4.0	18.2	3.3	68.4	9.0	1.1
Furfuryl alcohol	1.0	19.1	7.3	51.5	16.2	5.9
	2.0	16.2	6.1	59.8	14.4	3.5
	4.0	16.9	6.0	64.3	11.3	1.5
Hydroquinone	0.5	19.0	5.6	56.9	14.0	4.5
	1.0	19.0	5.4	58.2	12.8	4.6
	2.0	19.4	4.4	58.2	15.5	2.5
Catechol	0.5	21.8	5.5	59.4	12.5	0.8
	1.0	21.1	5.5	60.8	11.9	0.7
	1.5	18.4	6.1	60.2	14.8	0.5
Control		19.9	5.8	58.6	12.8	2.9

The toxicity of furfuryl alcohol was compared with its analogs furoic acid and furfural. Furoic acid showed less toxicity to the growth and lipid accumulation of *T. fermentans* [10]. Compared with the toxicity of furfural [11], furfuryl alcohol showed more serious inhibition on the growth and lipid accumulation of *T. fermentans* at low concentration (<10 mM). However, *T. fermentans* could suffer higher concentration of furfuryl alcohol than furfural. Similarly, the toxicity of vanillyl alcohol was compared with the vanillic acid, and vanillin. In spite of the fact that vanillyl alcohol showed stimulation on the lipid accumulation of *T. fermentans* at low concentration, the influence of vanillyl alcohol on the growth and lipid accumulation was greater than vanillic acid [10], but less than that of vanillin [11].

Besides the effect of individual alcohol compound, it is worth noting that most binary combination of alcohol compounds didn’t show synergistic inhibitory effect on the growth and lipid accumulation of *T. fermentans*. However, the binary combination of catechol and vanillyl alcohol or hydroquinone showed strong synergistic inhibition that even completely inhibited the growth of *T. fermentans* (Fig. S1).

The effect of alcohol compounds on the fatty acid composition of lipid from *T. fermentans* was shown in Table 2. Apparently, oleic acid was the most abundant one, being about 50–60% of the total fatty acids, followed by palmitic acid, stearic acid and linoleic acid. In most cases, alcohol compounds had no obvious influence on the composition of unsaturated acids, including oleic acid and linoleic acid. Interestingly, furfuryl alcohol showed certain impact on the lipid composition of lipid from *T. fermentans* that the ratio of oleic acid in its lipid increased as the concentration of furfuryl alcohol increased.

**Figure 2:**
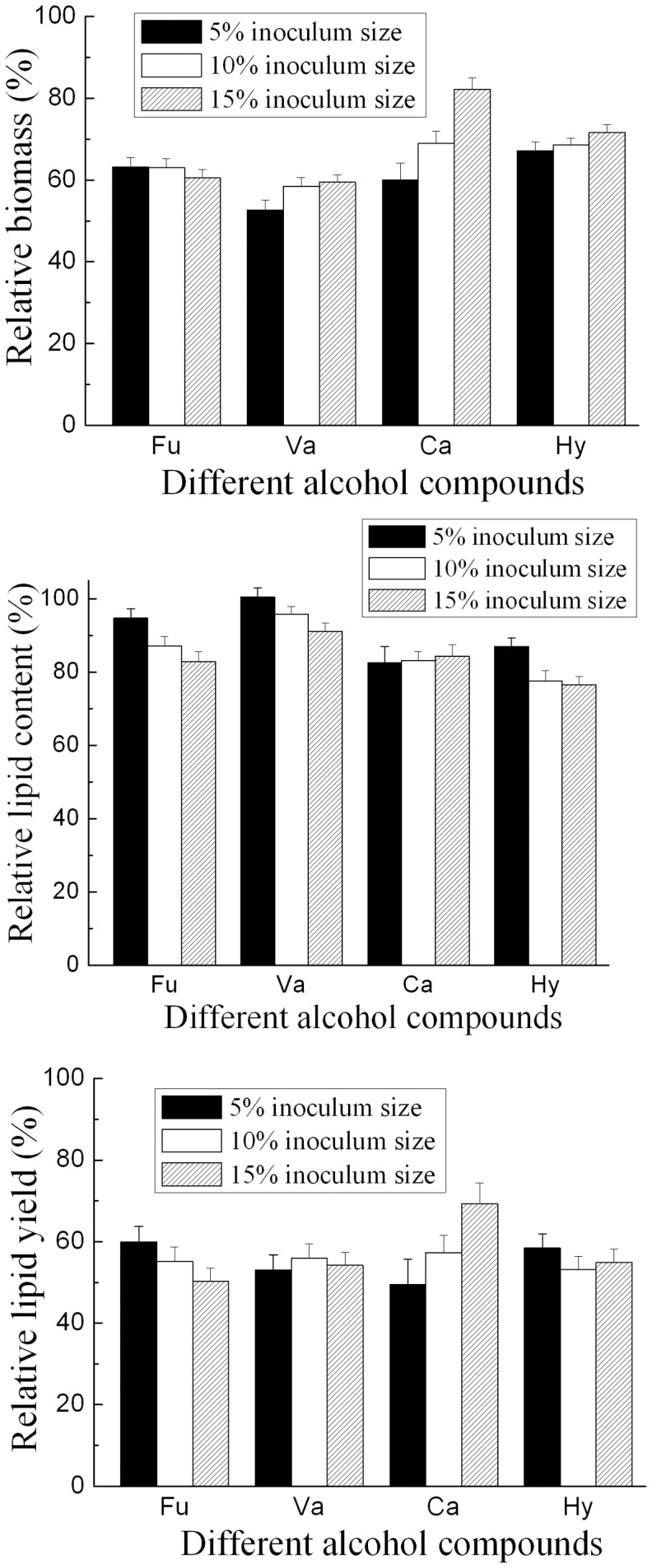
Effect of inoculum size on the inhibition of alcohol compounds. Each alcohol compounds was tested at its concentration of IC_50_. Cultures were incubated at initial pH 6.5, 25°C and 160 rpm for 7 days. Results are expressed relative to controls without organic acids. Biomass, lipid content and lipid yield of cultures in the absence of organic acids with 5%, 10% and 15% inoculum size were 24.0 g/L, 61.7% and 14.8 g/L; 22.4 g/L, 58.6% and 13.1 g/L; 21.6 g/L, 54.3% and 11.7 g/L, respectively. Abbreviations: Ca, catechol; Hy, Hydroquinone; Fu, Furfuryl alcohol; Va, Vanillyl alcohol.

### Effects of Inoculum Size, Temperature, and Initial pH on the Inhibition of Alcohol Compounds

In order to overcome the inhibitory effect of inhibitors in lignocellulosic hydrolysates, detoxification including physical, chemical and biological pretreatment was necessary for the lipid fermentation. Albeit these methods have been explained in details [5], [17]–[19], all of them would unfortunately increase the cost of SCO production. However, it is also proven that merely change the fermentation condition such as inoculum size, temperature, and initial pH value could mitigate the inhibitory effect [7], [12]. For example, in the studies on the ethanologenic bacteria *E. coli*, raising inoculum size and changing fermentation condition such as cultural temperature or initial pH have been considered as a strategy to decrease the inhibitory effect of alcohol compounds, aldehydes and organic acids [13]–[15]. In our recent works on oleaginous yeast *T. fermentans*, this strategy is also effective for overcoming the inhibition of aldehydes and organic acids [10], [11]. Thus, the effect of inoculum size, temperature, and initial pH on the inhibition of alcohol compounds on the growth and lipid accumulation of *T. fermentans* was investigated.

**Table 3:** Influence of fermentation factors on the inhibition of different inhibitors.

Inhibitors		Influence		
		Inoculum size	Temperature	Initial pH
Organic acids a	Formic acid	+ c	− e	− h
	Acetic acid	+	– f	–
	Levulinic acid	+	–	+ i
	4-Hydroxybenzoic acid	− d	–	+
	Vanillic acid	–	–	+
	Syringic acid	–	+ g	–
	Furoic acid	–	–	+
	Gallic acid	–	+	–
	Ferulic acid	–	–	+
	Caproic acid	+	+	– j
Aldehydes b	Furfural	+	–	+
	HMF	+	–	+
	4-Hydroxybenzaldehyde	+	+	–
	Syringaldehyde	+	+	–
	Vanillin	+	+	–
Alcohol compounds	Catechol	+	–	–
	Hydroquinone	–	–	–
	Furfuryl alcohol	–	–	+
	Vanillyl alcohol	–	–	–

a Base on the results of our previous work [10].

b Base on the results of our previous work [11].

c Inhibition could be relieved by higher inoculum size.

d Inhibition could not be relieved by higher inoculum size.

e Inhibition was more serious at higher temperature.

f Inhibition varied with different temperature.

g Inhibition was more serious at lower temperature.

h Inhibition was more serious at higher initial pH value.

i Inhibition was more serious at lower initial pH value.

j Inhibition varied with different initial pH value.

As shown in Fig. 2, greater inoculum size could reduce the inhibitory effect of all the alcohol compounds on the growth of *T. fermentans* except furfuryl alcohol. However, greater inoculum size could merely overcome the inhibition of catechol on the lipid accumulation of *T fermentans*, and in the medium containing other alcohol compounds tested, the relative lipid content of *T fermentans* decreased as the increase of inoculum size. This result was further compared with its effect on the inhibition of organic acid [10] and aldehyde [11]. As shown in Table 3, the effect of inoculum size on the inhibition of alcohol compounds was similar to our previous work focused on the effect of organic acids on the growth and lipid accumulation of *T. fermentans* [10]. The inhibition of aliphatic acids could be alleviated by greater inoculum size while the inhibition of aromatic or furan acids could not. However, greater inoculum size could reduce the inhibitory effect of the aldehydes [11]. It has been reported that aldehydes could be converted into other low toxic compounds during the fermentation process and higher inoculum size could boost the efficiency of this *in vivo* detoxification [12]. It might be due to no similar bioconversion was existed for most alcohol compounds, so greater inoculum size was not effective for overcoming the inhibition of alcohol compounds.

**Figure 3:**
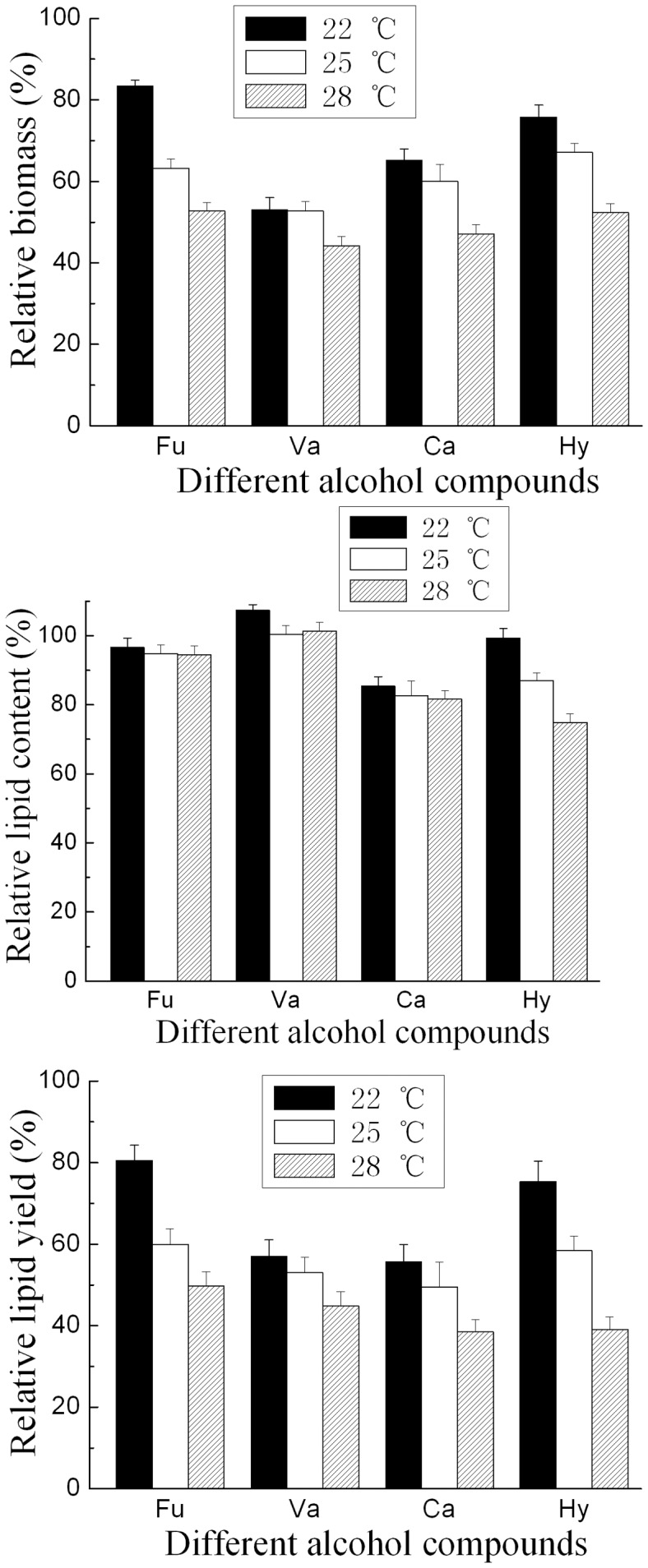
Effect of temperature on the inhibition of alcohol compounds. All alcohol compounds were tested at their respective concentration of IC_50_. Cultures with 5% inoculum size were incubated at initial pH 6.5 and 160 rpm for 7 days. Results are expressed relative to controls without organic acids. Biomass, lipid content and lipid yield of cultures lacking organic acids at 22°C, 25°C and 28°C were 19.9 g/L, 55.8% and 11.1 g/L; 24.0 g/L, 61.7% and 14.8 g/L; 23.6 g/L, 58.9% and 13.9 g/L, respectively. Abbreviations: Ca, catechol; Hy, Hydroquinone; Fu, Furfuryl alcohol; Va, Vanillyl alcohol.

**Figure 4:**
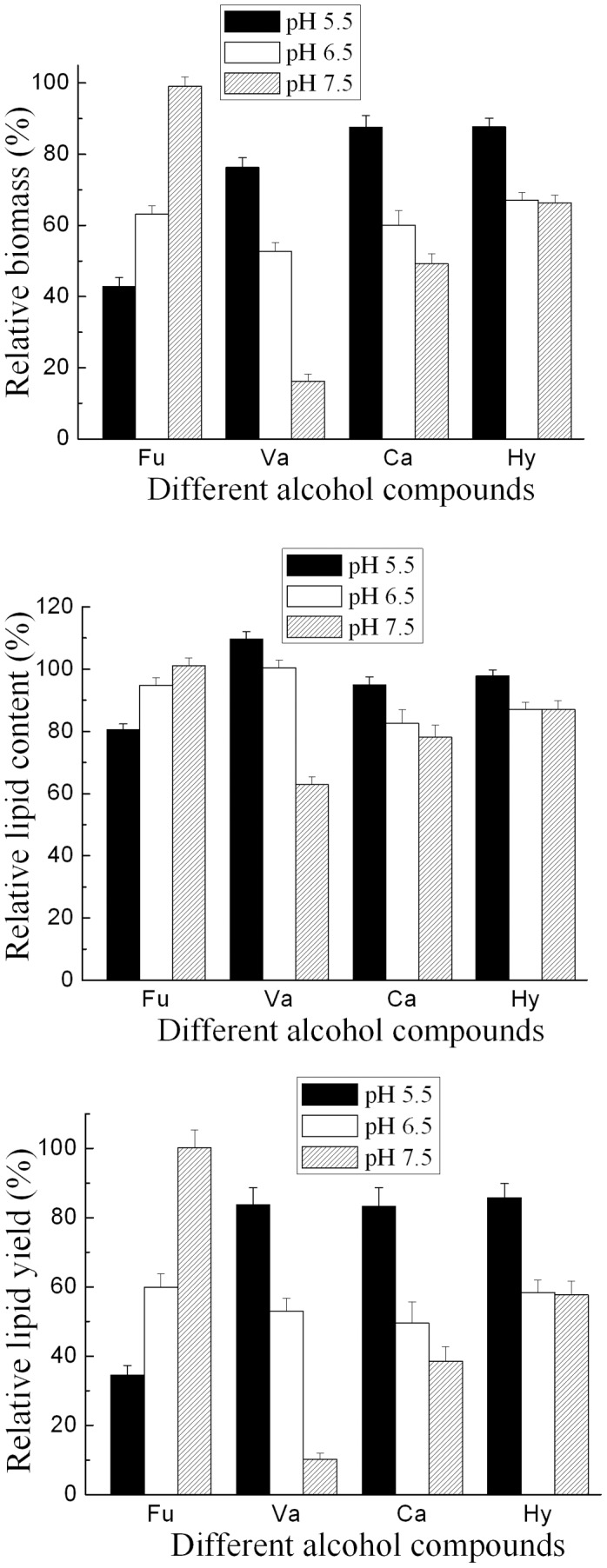
Effect of initial pH on the inhibition of alcohol compounds. All alcohol compounds were tested at their respective concentration of IC_50_. Cultures with 5% inoculum size were incubated at 25°C and 160 rpm for 7 days. Results are expressed relative to controls without organic acids. Biomass, lipid content and lipid yield of cultures lacking organic acids at pH 5.5, pH 6.5 and pH 7.5 were 18.4 g/L, 57.2% and 10.5 g/L; 24.0 g/L, 61.7% and 14.8 g/L; 21.5 g/L, 56.3% and 12.1 g/L, respectively. Abbreviations: Ca, catechol; Hy, Hydroquinone; Fu, Furfuryl alcohol; Va, Vanillyl alcohol.

Besides the effect of inoculum size, the effect of initial pH and temperature on the growth and lipid accumulation of *T. fermentans* was also investigated (Fig. 3 and Fig. 4). As shown in Fig. 3, both relative biomass and relative lipid content of *T. fermentans* increased as the decrease of temperature, suggesting that lower temperature could reduce the toxicity of all these four alcohol compounds on the growth and lipid accumulation of *T. fermentans*. Fig. 4 depicted the effect of initial pH on the inhibition of alcohol compounds. Lower initial pH could decrease the inhibitory effect of all the alcohol compounds except furfuryl alcohol on both the growth and lipid accumulation of *T. fermentans*. On the other hand, in the medium containing furfuryl alcohol, higher initial pH was better for the growth and lipid accumulation of *T. fermentans*. Overall, initial pH value influenced more dramatically than inoculum size and cultural temperature on the lipid fermentation of *T. fermentans*. We further examined the pH change during the fermentation and found that the pH decreased as the fermentation went on in both the media with or without inhibitor (e.g. the pH value would decrease from 6.5 to 3.8 after 7 days’ fermentation in the medium with no inhibitor). Carrying out fermentation at the optimal pH condition might be beneficial for the growth and lipid accumulation of *T. fermentans*. However, the biomass and lipid content of *T. fermentans* was not greater in the medium with buffer capacity (0.1 mol/L citric acid-citric acid sodium buffer, pH 6.5) than that in the medium without buffering conditions. On the other hand, the growth and lipid accumulation of *T. fermentans* could be stimulated in the medium containing acetic acid (this medium had certain buffer capacity) as mentioned in our previous work [10]. Base on the above-mentioned results, controlling pH by suitable buffer solution or mode, such as feeding sodium hydroxide or hydrochloric acid into fermenter might help improving lipid yield of *T. fermentans*, but it still needs to be further investigated.

In Table 3, the effect of cultural temperature and initial pH was further compared with their effect on the inhibition of both aldehydes and organic acids. It is worth noting that initial pH showed more significant influence on the inhibitory effect of organic acids [10] than those of aldehydes [11] and alcohol compounds. It is proven that organic acid might affect intracellular pH value and thus changing the physiology of microorganisms [20]–[22].

All in all, the cell growth of *T. fermentans* was more sensitive to the variation of inoculum size, temperature, and initial pH than lipid synthesis in the presence of alcohol compounds.

**Figure 5:**
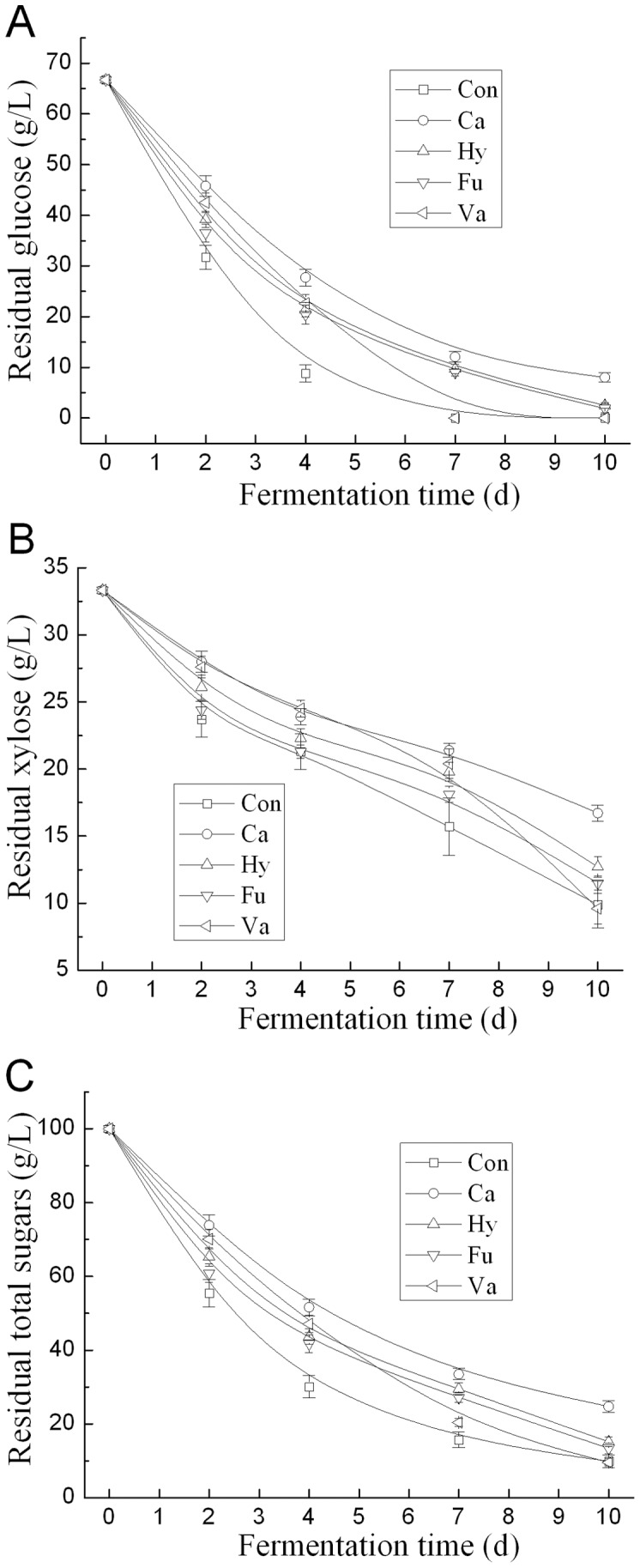
Effect of the selected alcohol compounds on the sugar metabolism of *T. fermentans*. (A) Glucose consumption; (B) Xylose consumption; (C) Total sugars consumption. The residual glucose, xylose, and total sugars in the control medium at the 2^nd^, 4^th^, 7^th^ and 10^th^ day were (g/L) 31.7, 23.7, 55.4; 8.8, 21.3, 30.1; 0, 15.7, 15.7; and 0, 9.9, 9.9, respectively. Abbreviations: Ca, catechol; Hy, Hydroquinone; Fu, Furfuryl alcohol; Va, Vanillyl alcohol; Con, Control.

### Inhibitory Mechanisms of Alcohol Compounds on the Growth and Lipid Accumulation of *T. fermentans*

The mechanisms of inhibitory effect of inhibitors present in lignocellulosic hydrolysates on the growth and metabolism of ethanologenic yeasts have been systematically summarized in previous reviews [7], [12]. However, little has been known about the inhibitory mechanism on the oleaginous microorganisms. In many cases, the sugar utilization could be inhibited by the inhibitors. For example, furan aldehydes would influence the activity of the key enzyme in the glycolysis and tricarboxylic acid cycle (TCA), and thus elongating the lag phase of microorganisms [23]. On the other hand, in our previous works, it is shown that the key enzyme (malic enzyme) activity of lipid accumulation might be inhibited by aldehydes and organic acids [10], [11]. Moreover, some inhibitors such as aromatic compounds would attack the hydrophobic sites on the cell membrane and thus destroy its integrity [24]. Also, it is reported that these inhibitors could also influence the cell morphology [25]. For the inhibition of alcohol compounds on the ethanologenic *E. coli*, the inhibitory mechanisms mainly include the inhibition on the sugar metabolism and the damage on the cell membrane [7]. Base on the discussion above, the effect of selected alcohol compounds on the sugar utilization, malic enzyme activity, cell membrane integrity, and cell morphology was measured in order to preliminarily elucidate the inhibitory mechanisms of alcohol compounds on the growth and lipid accumulation of *T. fermentans*.

**Figure 6:**
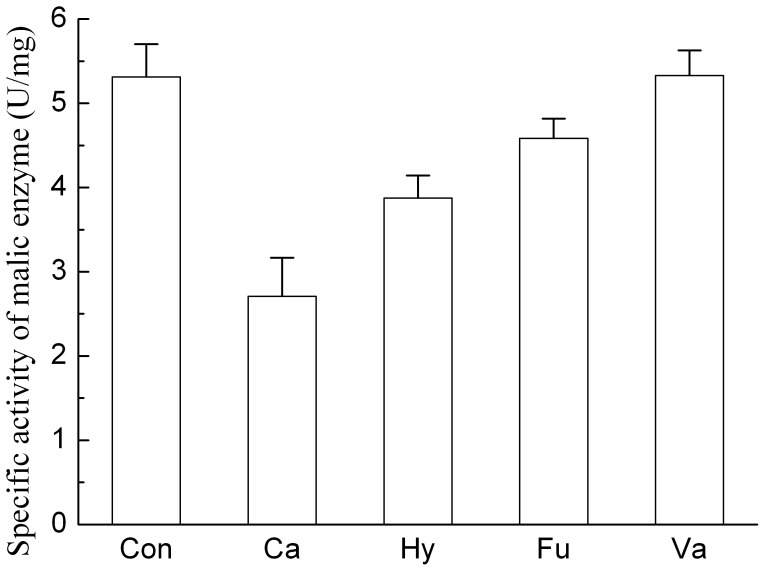
Effects of alcohol compounds on the malic enzyme activity of *T. fermentans.* Biomass cultivated in the medium with and without inhibitor was harvested and disrupted to get the crude enzyme. Then the malic enzyme activity was measured and compared. Abbreviations: Ca, catechol; Hy, Hydroquinone; Fu, Furfuryl alcohol; Va, Vanillyl alcohol; Con, Control.

The effect of alcohol compounds on the sugar utilization of *T. fermentans* was tested at their IC_25_ concentration. As shown in Fig. 5, the utilization of both glucose and xylose were inhibited by these four alcohol compounds and the inhibition by catechol was more serious than other three ones. The inhibition of alcohol compounds on both glucose and xylose metabolism partly accounted for the inhibition of alcohol compounds on the growth and lipid accumulation of *T. fermentans*. Interestingly, after 7 days’ fermentation, vanillyl alcohol showed some stimulation on xylose utilization of *T. fermentans*.

**Table 4:** Influence of different kinds of inhibitors on the xylose utilization, malic enzyme activity, and cell morphology of *T. fermentans.*

Inhibitors		Different effect		
		Xylose utilization	Malic enzyme activity	Cell morphology
Organic acids a	Formic acid	− c	− e	+ g
	Acetic acid	–	–	− h
	Levulinic acid	–	–	–
	4-Hydroxybenzoic acid	–	–	–
	Vanillic acid	+ d	–	–
	Syringic acid	+	–	+
	Furoic acid	+	+ f	–
	Gallic acid	–	–	+
	Ferulic acid	+	–	+
	Caproic acid	–	–	–
Aldehydes b	Furfural	+	–	+
	HMF	+	+	–
	4-Hydroxybenzaldehyde	+	–	–
	Syringaldehyde	+	–	+
	Vanillin	+	+	+
Alcohol compounds	Catechol	–	–	+
	Hydroquinone	–	–	+
	Furfuryl alcohol	–	–	+
	Vanillyl alcohol	+	– i	+

a Data about xylose utilization and malic enzyme activity were base on our previous work [10].

b Data about xylose utilization, malic enzyme activity, and cell morphology base on our previous work [11].

c No stimulation on xylose utilization of *T. fermentans.*

d Stimulation on xylose utilization of *T. fermentans.*

e No influence on malic enzyme activity of *T. fermentans.*

f Stimulation on malic enzyme activity of *T. fermentans.*

g Influence on cell morphology of *T. fermentans.*

h No influence on cell morphology of *T. fermentans.*

i Little influence on malic enzyme activity of *T. fermentans.*

Besides the unblocked sugar metabolism, lipid accumulation of oleaginous microorganisms required a great amount of reduced force (NAPDH) for the synthesis of fatty acids. Thus, malic enzyme was considered as one of the most important enzyme in the pathway of lipid synthesis [26]. The effect of the selected alcohol compounds on the malic enzyme activity of *T. fermentans* at their respective IC_25_ concentrations were tested after the 2^nd^ day of fermentation when the lipid formation rate reached the maximal in the control culture without inhibitors. As can be seen in Fig. 6, all the four alcohol compounds except vanillyl alcohol showed inhibitory effect on the malic enzyme activity, which partly explained the inhibitory effect of alcohol compounds on the lipid accumulation of *T. fermentans*. Interestingly, the malic enzyme activity of *T. fermentans* was affected little by vanillyl alcohol, which partly explain the phenomenon that it could stimulate the lipid accumulation of *T. fermentans* at its low concentration (<25 mM, Fig. 1).

**Figure 7:**
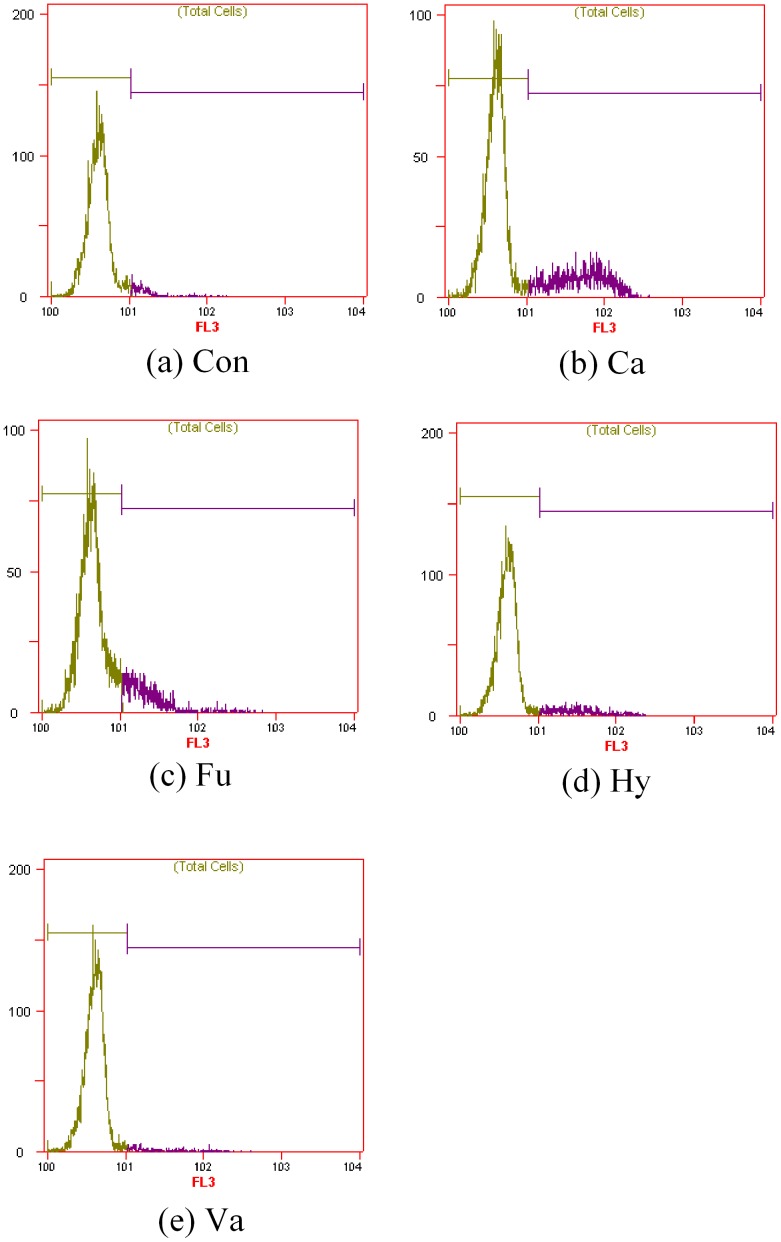
Effects of alcohol compounds on the cell membrane integrity of *T. fermentans*. X-axe: signal intensity; Y-axe: number of cells/mL (×1000). Cells of *T. fermentans* in the medium with and without inhibitor were stained at the same condition and then stained cells were analyzed by flow cytometry. Abbreviations: Ca, catechol; Hy, Hydroquinone; Fu, Furfuryl alcohol; Va, Vanillyl alcohol; Con, Control.

**Figure 8:**
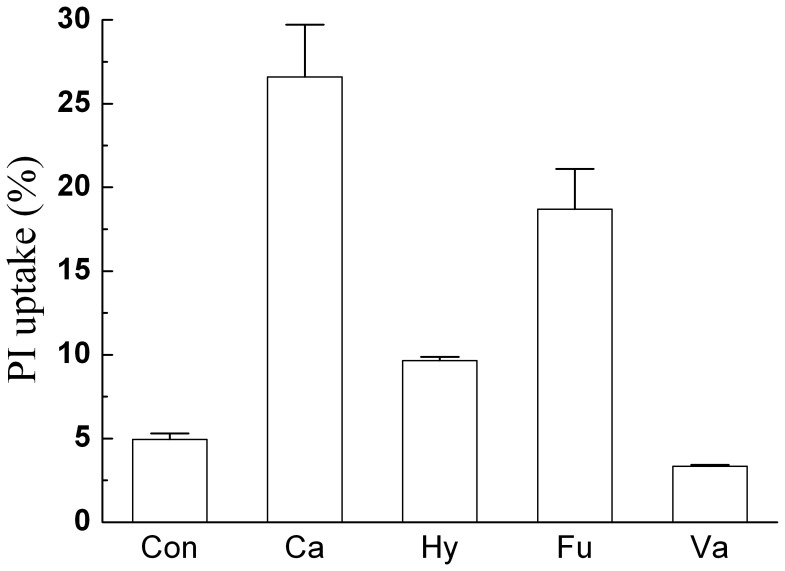
PI uptake of *T. fermentans*’s cells in the medium containing different alcohol compounds. Cells of *T. fermentans* in the medium with and without inhibitor were stained at the same condition and then stained cells were analyzed by flow cytometry. The PI uptake was calculated by Cell Lab Quanta SC software. Abbreviations: Ca, catechol; Hy, Hydroquinone; Fu, Furfuryl alcohol; Va, Vanillyl alcohol; Con, Control.

In our previous works focused on the effect of organic acids and aldehydes on the growth and lipid accumulation of *T. fermentans*, many inhibitory compounds showed stimulation on the xylose metabolism of *T. fermentans*. These results were further summarized and compared in Table 4. As it depicted, most compounds such as vanillin, vanillic acid, vanillyl alcohol and etc. which could stimulate the xylose utilization of *T. fermentans* were aromatic ones, indicating that phenyl structure might stimulate the enzyme activity of xylose consumption. Similar phenomenon was also observed by other researchers [27]. It is worth noting that in most cases, the increase in xylose utilization did not result in higher lipid production by *T. fermentans*. The effect of alcohol compounds on the malic enzyme activity was also further compared with the effect of organic acids [10] and aldehydes [11]. As shown in Table 4, all the inhibitors expect furoic acid, vanillin, and HMF showed inhibitory effect on the malic enzyme activity of *T. fermentans*. In our previous study [10], furoic acids showed similar stimulation on the lipid accumulation of *T. fermentans* as vanillyl alcohol did. Its enhancement in malic activity of *T. fermentans* might also account for this. However, in the medium containing vanillin or HMF, no stimulation on lipid accumulation of *T. fermentans* was observed [11].

The morphological changes of *T. fermentans* were observed microscopically during cultivation in the presence of alcohol compounds at their concentrations of IC_50_ (Fig. S2). The cell morphology of *T. fermentans* on the medium without inhibitors was typically elliptical. In the presence of most alcohol compounds involving catechol, hydroquinone, and furfuryl alcohol, cells appeared elongated as rods or short chains during fermentation. However, the cell morphology of *T. fermentans* was round-like in the medium containing vanillyl alcohol. The effect of alcohol compounds on the cell morphology was further compared with the effect of organic acids and aldehydes (Table 4). The data in Table 4 further proved that many inhibitors could change the cell morphology of *T. fermentans*. It is well known that the inhibitors present in the lignocellulosic hydrolysates would change the cell morphology of microorganisms [13]–[15]. However, it has also been reported that there is no obvious relationship between the difference of cell morphology and the inhibition on the growth of microorganisms [25], as indicated in this work.

It has been reported that aromatic compounds could damage the cell membrane integrity and influence the physiology of microorganisms [12]. Hence, the effect of alcohol compounds at their IC_50_ concentration on the cell membrane integrity of *T. fermentans* was investigated after two days’ fermentation. As shown in Fig. 7 and Fig. 8, all the alcohol compounds except vanillyl alcohol showed certain damage on the cell membrane integrity and more serious damage on the cell membrane integrity it did, greater PI uptake rate it had. Among the four alcohol compounds tested, catechol showed the most serious damage on the cell membrane integrity of *T. fermentans*, following by furfuryl alcohol and hydroquinone. Interestingly, vanillyl alcohol had little influence on the cell membrane integrity of *T. fermentans*, which was in good accordance with previous work on ethanologenic bacteria *E. coli* [13]. It is worth noting that the hydrophobicity of vanillyl alcohol and furfuryl alcohol was similar (as indicated by log *P* value), but furfuryl alcohol showed much greater damage on the cell membrane integrity of *T. fermentans* than vanillyl alcohol, partly explained the phenomenon mentioned above that the toxicity of alcohol compounds to *T. fermentans* was not related to their hydrophobicity.

### Conclusions

The toxicity of alcohol compounds to *T. fermentans* varied with their structures and concentrations. The toxicity of alcohol compounds except furfuryl alcohol was well related to the log *P* value. Inoculum size and environmental factors (temperature and initial pH of medium) also had influences on the inhibition of alcohol compounds, and the inhibition could be partly relieved by careful control of the culture conditions. Thus, optimization of fermentation conditions, such as maintaining pH at the optimal status by feeding sodium hydroxide or hydrochloric acid into fermenter could be helpful for improving lipid yield of *T. fermentans* in lignocellulosic hydrolysates. Most alcohol compounds showed inhibition on sugar consumption and malic enzyme activity of *T. fermentans*. Similarly, all alcohol compounds except vanillyl alcohol exerted damage on the cell membrane of *T. fermentans*. The results achieved here show that the inhibitory mechanisms of inhibitors on *T. fermentans* are related with their effects on sugar consumption, key enzyme activity and cell membrane integrity. Adoption of domestication and/or biotechnological approaches, such as genetic engineering to enhance the inhibitor-resistant ability of *T. fermentans* is potential to improve the efficiency of lipid production in lignocellulosic hydrolysates.

## Materials and Methods

### Microorganism and Chemicals

Oleaginous yeast *Trichosporon fermentans* CICC 1368 was obtained from the China Center of Industrial Culture Collection and kept on wort agar at 4°C.

Catechol, hydroquinone, furfuryl alcohol, and vanillyl alcohol were obtained from Alfa Aesar (UK). All other chemicals were from commercial sources and were of the highest purity available.

### Medium, Precultivation and Cultivation

The precultivation medium contained (g/L): glucose and xylose (ratio 2∶1) 20, peptone 10, yeast extract 10, pH 6.0. And the fermentation medium included (g/L): glucose and xylose (ratio 2∶1) 100, yeast extract 0.5, peptone 1.8, MgSO_4_·7H_2_O 0.4, KH_2_PO_4_ 2.0, MnSO_4_·H_2_O 0.003, CuSO_4_·5H_2_O 0.0001, pH 6.5. The preculture was performed in a 250 mL conical flask containing 50 mL precultivation medium in a rotary shaker at 28°C and 160 rpm for 24 h. Then, 5% seed culture (2.5 mL) was inoculated to a 250 mL conical flask containing 47.5 mL fermentation medium and cultivation was carried out in a rotary shaker at 25°C and 160 rpm for 7 days.

To facilitate averaging, results are expressed as a percentage of the control value without adding the tested inhibitor.

### Effect of Alcohol Compounds on Growth and Lipid Accumulation

After precultivation, 2.5 mL seed culture was inoculated to 47.5 mL fermentation medium containing various alcohol compounds. In the absence of an inhibitor, biomass, lipid content, lipid yield, and residual sugar concentration after 7 days’ fermentation were 24.0 g/L, 61.7%, 14.8 g/L, and 15.7 g/L, respectively. IC_25_ and IC_50_, defined as concentrations of the test alcohol compounds that caused 25% or 50% inhibition on the lipid yield, respectively, were measured on the data shown in Fig. 1. All reported data were averages of experiments performed at least in duplicate.

### Effect of Inoculum Size, Temperature and Initial pH on the Inhibition of Alcohol Compounds

The effect of inoculum size on the potency of alcohol compounds was examined using alcohol compound concentrations of IC_50_. 5%, 10% and 15% seed culture were inoculated to the fermentation media containing various alcohol compounds respectively.

The effect of temperature on the potency of alcohol compounds was studied using alcohol compound concentrations of IC_50_. The cultures with 5% inoculum size were maintained at 22°C, 25°C, and 28°C respectively.

The effect of initial pH on the potency of alcohol compounds was investigated using alcohol compound concentrations of IC_50_. Fermentation media containing alcohol compounds were adjusted to either pH 5.5, 6.5, or 7.5 prior to inoculation. The variation of pH was measured periodically throughout the fermentation process. Moreover, the lipid fermentation was carried out in the medium with buffer capacity (0.1 mol/L citric acid-citric acid sodium buffer, pH 6.5) and compared with that in the medium without buffer capacity as described above.

Biomass, lipid content, and lipid yield were all measured after 7 days’ fermentation.

### Binary Combinations of Alcohol Compounds

Two selected alcohol compounds with each concentration being IC_25_ were added to the fermentation medium. Cultures were inoculated as described above and incubated for 7 days (5% inoculum size, pH 6.5 and 25°C). Cultures grown without alcohol compounds were included as controls.

### Effect of Alcohol Compounds on Sugars Utilization

The sugar consumption was tested by using alcohol compound concentrations of IC_25_. The relative sugar consumption is defined as the ratio of the amount of glucose and xylose consumed by the yeast cells grown in the culture medium containing various alcohol compounds for 7 days to that without inhibitors.

### Effect of Alcohol Compounds on Malic Enzyme Activity

Biomass was harvested by centrifugation (4,000 g for 15 min at 4°C), washed three times with distilled water and then suspended in extraction buffer (50 mM Tris-HCl containing 1 mM MgCl_2_ and 1 mM DTT, pH 7.5). After being disrupted by a sonic cell disrupter at 300 W for 10 min on ice, the mixture was centrifuged (10,000 g for 10 min at 4°C) and the supernatant was used immediately for enzyme activity assay.

Malic enzyme activity was detected at 340 nm and 30°C with a SHIMADZU UV-2550 spectrophotometer (Japan). The reaction was initiated by adding 10 mM sodium L-malate into 3 mL reaction mixture containing 50 mM Tris-HCl (pH 7.5), 10 mM MgCl_2_, 0.5 mM NADP^+^, and 0.04 mL cell extract. The absorbance of the formed NADPH was recorded after reaction at 30°C for 3 min. A control experiment, which was performed by following the above procedure except that no sodium L-malate was added, demonstrated that no NADP^+^-NADPH conversion was detectable. One unit corresponds to the amount of enzyme producing 1 nmol NADPH per minute at 30°C.

### Flow Cytometry (FCM)

Cell samples were diluted to approximately 10^6^ cells/mL and processed for sorting at a rate of about 500 events per second. In total, 10,000 events were detected each run. Flow cytometry coupled with propidium iodide (PI) (KeyGEN, China) was used in this study in order to monitor *T. fermentans* cytoplasmic membrane integrity. PI binds to DNA and cannot cross an intact cytoplasmic membrane. Samples taken from the culture were immediately diluted with phosphate buffer solution (PBS, pH 7.0) and stained with PI.

Flow cytometry was carried out using a Cell Lab Quanta™ SC instrument (Beckman Coulter, USA) fitted with a 22 mW ion laser for excitation (488 nm) while monitoring with a single emission channel (575-nm band-pass filter). Cell Lab Quanta SC software (Beckman Coulter, USA) was used for instrument control, data acquisition and data analysis.

### Analytical Methods

Biomass was harvested by centrifugation and weighed in its lyophilized form [28]. Extraction of lipid from lyophilized biomass was performed according to the modified procedure of [29]. Lipid was extracted with a mixture of chloroform: methanol (2∶1, v/v) for 1 h. The extracted lipid was centrifuged to obtain a clear supernatant and the solvent was removed by evaporation under vacuum at 55°C and 100 rpm (NE-Series rotary evaporator EYELA, Japan). Lipid content was defined as the ratio of lipid weight to that of biomass.

The fatty acid profile of the lipid from *T. fermentans* was determined by gas chromatography (GC-2010) with an ionization detector and a DB-1 capillary column (0.25 cm × 30 m, Agilent Technologies Inc., USA) according to the published procedure (Zhu et al. 2008). D-Xylose and D-glucose were measured by HPLC (Waters Corp., USA) with a RI detector (Waters 2410) and an Aminex HPX-87P column (300 mm × 7.8 mm, Bio Rad Corp., USA) at 85°C. Deionized water was used as the mobile phase at 0.5 mL/min.

## Supporting Information

Figure S1 Effect of binary combinations of alcohol compounds on the growth and lipid accumulation of *T. fermentans.* Abbreviations: Ca, catechol; Hy, Hydroquinone; Fu, Furfuryl alcohol; Va, Vanillyl alcohol. ^a^ The cell biomass was hardly detected after fermentation
(TIF)

Figure S2 Effect of alcohol compounds on the cell morphology of *T. fermentans.* Abbreviations: Ca, catechol; Hy, Hydroquinone; Fu, Furfuryl alcohol; Va, Vanillyl alcohol; Con, Control.
(TIF)
