# Supplementary figures and images for: Effects of Alcohol Compounds on the Growth and Lipid Accumulation of Oleaginous Yeast Trichosporon fermentans
Source: PLoS One. 2012 Oct 5;7(10):e46975. doi: 10.1371/journal.pone.0046975 (PMC3465294; doi:10.1371/journal.pone.0046975)

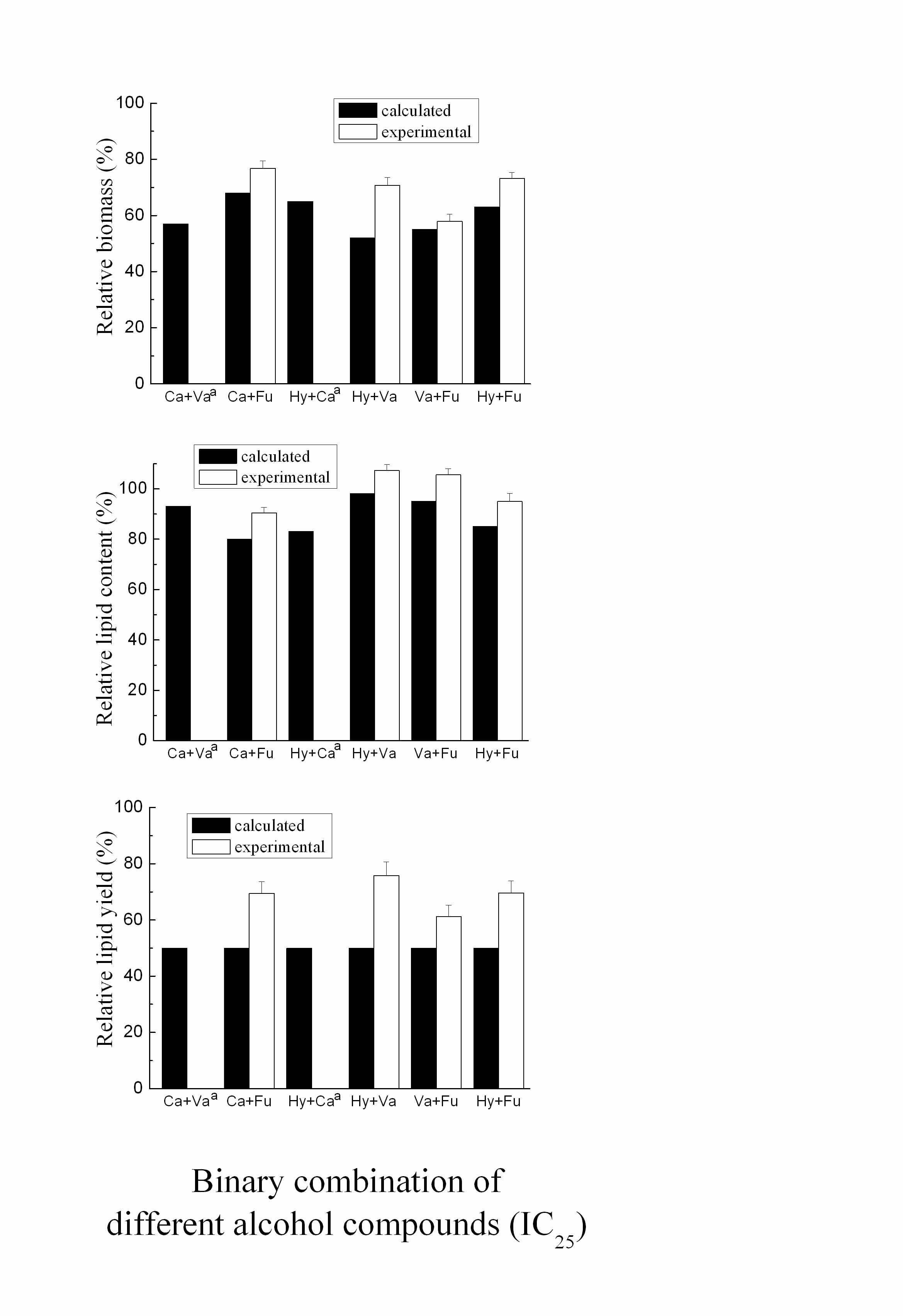

Supplement: Figure S1 — Effect of binary combinations of alcohol compounds on the growth and lipid accumulation of T. fermentans. Abbreviations: Ca, catechol; Hy, Hydroquinone; Fu, Furfuryl alcohol; Va, Vanillyl alcohol. a The cell biomass was hardly detected after fermentation (TIF) [file pone.0046975.s001.tif]

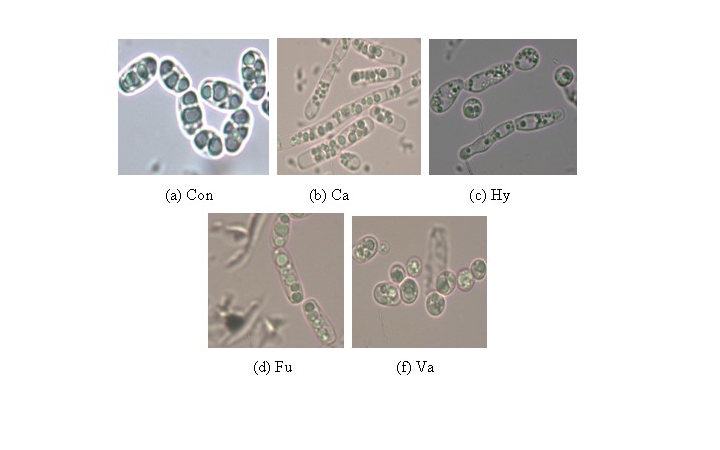

Supplement: Figure S2 — Effect of alcohol compounds on the cell morphology of T. fermentans. Abbreviations: Ca, catechol; Hy, Hydroquinone; Fu, Furfuryl alcohol; Va, Vanillyl alcohol; Con, Control. (TIF) [file pone.0046975.s002.tif]
